# Microvascular anatomy of the urinary bladder in the adult African clawed toad, *Xenopus laevis*: A scanning electron microscope study of vascular casts

**DOI:** 10.1002/jmor.21310

**Published:** 2020-12-25

**Authors:** Alois Lametschwandtner, Bernd Minnich

**Affiliations:** ^1^ Department of Biosciences University of Salzburg, Vascular and Exercise Biology Research Group Salzburg Austria

**Keywords:** microvascular anatomy, scanning electron microscopy, urinary bladder, vascular casts, Xenopus

## Abstract

We studied urinary bladders of adult male and female *Xenopus laevis* using light microscopy of stained tissue sections and scanning electron microscopy (SEM) of vascular corrosion casts (VCCs). Results showed that bilaterally a vesical artery branched off the femoral artery. At the dorso‐lateral serosal surface of the body of the bladder each artery splitted within a short distance into up to five smaller arteries that supplied body and neck regions. Arteries gave off short and long terminal arterioles, which fed the mucosal capillary meshwork. Long terminal arterioles followed dimensional changes of the bladder, while short ones anchored the capillary network to the arterial system. Capillary mesh sizes and shapes varied according to the filling state of the urinary bladder. In the highly to moderately distended (filled) bladder, capillaries were rather straight or undulated only slightly, in the contracted (emptied) bladder they undulated strongly and lay side by side. Postcapillary venules formed by two equally sized capillaries or from capillaries, which serially drained into a small postcapillary venule. Vesical venules formed a large dorsal vesical and a varying number of smaller lateral and ventral vesical veins. The dorsal vesical vein drained either directly or via the posterior hemorrhoidal vein into the common pelvic vein. Lateral and ventral vesical veins also drained into the latter. The vascular patterns found were discussed in respect to the bladder spatial movements during distention (filling) and relaxation (emptying). Furthermore, it was hypothesized that an extensively filled bladder could compress the overlaying abdominal vein forcing part of the blood otherwise drained towards the liver to be detoured via the renal portal veins to the kidneys.

## Graphical abstract

Lateral view at a vascular cast of the urinary bladder of adult *Xenopus laevis*. SEM micrograph. Slightly undulating branches of vesical arteries (va; red) indicate their capacity to straighten if distention (filling) of the bladder increases. Vesical veins (blue) are rather straight and adapt to a further increase in bladder volume by longitudinal stretching.

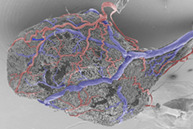

## 1 INTRODUCTION

The urinary bladder plays an important role in amphibian lifestyles. As anuran amphibians inhabit diverse environments, the amount of urine stored in their urinary bladders ranges from 50% of the body weight in the desert‐burrowing frogs *Notaden nicholsi* and *Neobatrachus wilsmorei* (Main & Bentley, 1964) to as little as 1% in the aquatic *Xenopus laevis* (Duellman & Trueb, 1994).

Different to the mammalian anatomy, where the urinary bladder fills via ureters, which directly empty into the bladder, in anurans ureters empty into the cloaca. From there, urine is backed up to the urinary bladder by cloacal smooth muscle contractions that have to overcome urinary bladder pressure resulting from bladder compliance, lung inflation, and buccal movements (Martin & Hillman, 2009).

Structure and function of the anuran urinary bladder are well described (see e.g. Bentley, 1966; Calamita et al., 1994; Gaupp, 1904; Mochida et al., 2008; Peachey & Rasmussen, 1961; Shibata et al., 2015; Wiechmann & Wirsig‐Wiechmann, 2003) as are gross arterial supply and venous drainage (Gaupp, 1904; Millard, 1940; Roth, 1973; Szarski, 1948). Millard (1940) found that in the African clawed toad, *Xenopus laevis*, a single vesical artery originated bilaterally from the femoral artery and approached the urinary bladder at its dorsal circumference (see her Figures 8 and 9). Her study was based on excellent dissections, but because of the limited depth of focus of the dissecting microscope she could not visualize the bladder microvascular anatomy and so studies on the microvascular anatomy of the anuran urinary bladder are still lacking. This strongly contrasts with the situation in mammals including humans, where we know the three‐dimensional microvascular anatomy of the healthy and diseased urinary bladder in detail (Hossler et al., 2013; Hossler & Kao, 2007; Hossler & Monson, 1995, 1998a, 1998b; Miodonski et al., 1998; Miodonski et al., 2001; Miodonski & Litwin, 1999).

The muscular layers allow amphibian urinary bladders for a great distention when they are filled with urine (Duellman & Trueb, 1994). We assumed that in such a situation a sufficiently high vesical blood flow can be only maintained if sufficient lengths of vesical vessels are present, which in the filled state should be rather straight or slightly undulating, but should highly undulate and be narrow spaced in empty bladders. To test this assumption, we analyzed vascular corrosion casts (VCCs) of urinary bladders of adult *Xenopus laevis* by the scanning electron microscope (SEM). The high depth of focus and a sufficiently high resolution gained at an acceleration voltage of 10 kV in the conventional scanning electron microscope allowed (i) to identify cast capillaries as the blood vessels with the thinnest diameter, (ii) to differentiate arteries and veins by their characteristic endothelial cell nuclei imprint patterns displayed on the surface of vascular casts (Miodonski et al., 1976), and (iii) to localize anatomical structures regulating blood flow in the bladder, i.e. arterial and venous sphincters (Aharinejad et al., 1991; Franz et al., 1990; Schraufnagel & Patel, 1990), flow dividers, intimal cushions (Bond et al., 2010; Casellas et al., 1982; Fourman & Moffat, 1961) and venous valves (Caggiati et al., 2006; Hossler & West, 1988).

Additionally, paraplast embedded, stained urinary bladder tissue sections were studied in order to attribute displayed vascular patterns in the fully digested urinary bladder cast specimens to defined tissue layers.

## 2 MATERIALS AND METHODS

### 2.1 Animals

Ten adult individuals of both sexes of the African Clawed Toad, *Xenopus laevis* (Daudin, 1802) were studied. Adults were housed in aquaria (tap water depth: 15 cm) equipped with aquarium filters and fed twice a week with either dried *Gammarus pulex* or ground beef heart.

### 2.2 Histomorphology

Two animals (female: body weight: 79 grams, body length: 90 mm; male: body weight: 79 grams, body length: 90 mm) were euthanized by immersion into an 0.5% aqueous solution of MS 222 (Tricaine methanesulfonate; Sigma‐Aldrich Chemie, Steinbuch, Germany). After weighing, the heart was exposed, the sinus venosus opened and animals were perfused with amphibian Ringer's solution (Adam & Czihak, 1964) to remove blood and fixed with Bouin's solution via the truncus arteriosus. The flow rate of the infusion pump (Habel, Vienna) was set to 41 mL/h. Fixed urinary bladders were excised, dehydrated in a graded series of ethanol, and embedded in paraplast. Transverse sections (thickness: 7 μm) were stained with Goldner's trichrome (Adam & Czihak, 1964) and analyzed with an Olympus BX51 light microscope. Images were recorded by a digital camera (Olympus SC 50; Japan) attached to the microscope using Cell Sense Imaging software (Olympus, Japan). If necessary, brightness and contrast of images was adjusted using Photoshop 7.0 (Adobe Inc., Redwood, CA).

### 2.3 Vascular corrosion casting

Eight adult animals (5 males: body weights: 19–39 grams, body lengths: 80–90 mm: 3 females: body weights: 19–39 grams, body lengths: 60–75 mm) were studied. Euthanasia and rinsing perfusion were carried out in an identical manner to the histological study. When clear amphibian's Ringer's solution drained from the opened sinus venosus, 10 mL of Mercox CL‐2B (Dainippon Ink and Chemicals, Tokyo, Japan; Ladd Burlington, Vermont, USA) diluted with monomeric methylmethacrylate (4 + 1, v + v, 10 mL monomeric methylacrylate contained 0.85 g initiator paste MA) were injected with the infusor at a flow rate of 41 mL/h. When the effluent resin became more viscous or the whole amount of resin had been perfused, the injection was stopped, and the animals were left for about 30 minutes at room temperature to allow hardening of the injected resin. After tempering the injected resin by submerging the whole animal into a water‐bath (60°C; 12–24 hours), specimens were macerated in potassium hydroxide (7.5%; 40°C; 2–24 h). Thereafter rinsed three times in distilled water, submerged in 2% hydrochloric acid, rinsed again three times in distilled water, and submerged in formic acid (5%; 20°C; 5–15 minutes) to remove any residual organic matter from the cast surfaces. Finally, specimens were rinsed for another three times in distilled water and frozen in fresh distilled water. Ice‐embedded casts were freeze‐dried in a Lyovac GT2 (Leybold‐Heraeus, Cologne, DE). Casts of the abdominal area with the urinary bladder in‐situ were mounted dorsal side down onto specimen stubs using the “conductive bridge‐method” (Lametschwandtner et al., 1980). Mounted specimens were either evaporated with carbon and gold or sputter‐coated with gold and examined in the scanning electron microscope XL‐30 (FEI, Eindhoven, The Netherlands) at an accelerating voltage of 10 kV.

After a first SEM‐inspection, course, branching patterns and areas of supply (or drainage) of individual abdominal and urinary bladder vessels were exposed in some specimens by ripping‐off overlaying vessels with fine tipped insect pins under binocular control. Specimens then were sputter‐coated with gold and analyzed in the SEM.

To allow less experienced readers to easily distinguish arteries from veins in SEM micrographs we color‐coded arteries (red) and veins (blue) by using Photoshop 7.0 (Adobe Inc., Redwood, CA, US).

The study was approved by the Ethics Committee of the University of Salzburg, Austria and the Federal Government (BMBWK‐66.012/0018‐BrGT/2006).

## 3 RESULTS

**FIGURE 1:**
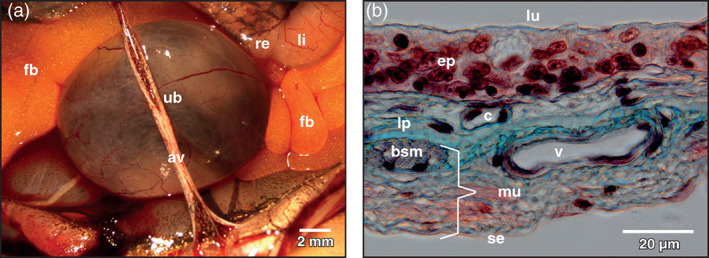
*Xenopus laevis*, urinary bladder (ub) of an adult individual. (a) Ventral view at the spherical body of the exposed organ. Neck region is hidden. Stereomicroscopy. av abdominal vein, fb fat body, li large intestine, re rectum, ub urinary bladder. (b) Light microscopic structure of the wall of the bladder. Detail view. Paraplast embedded tissue section (7 μm). Goldner's trichrome stain. Note the comparatively thick transitional epithelium (ep). bsm bundle of smooth muscle cells, c capillary, lp lamina propria, lu lumen of bladder, mu muscularis, se serosa, v vein

The moderately filled urinary bladder of adult *Xenopus laevis* had an ovoid to spherical body (Figure 1(a)) and a funnel‐like neck, which connected to the ventral portion of the cloaca. Its wall (from luminal to abluminal) consisted of the mucosa composed of a transitional epithelium and a lamina propria with blood vessels and loose connective tissue, the muscular layer with scattered, interwoven bundles of smooth muscle cells arranged circularly and longitudinally, and the serosa made up of squamous mesothelial cells (Figure 1(b)).

### 3.1 Vascular anatomy

In only one (out of eight) specimen an almost perfect filling with few missing cast microvessels was gained. In all other specimens filling of vascular beds ranged from good to poor but allowed the recognition of individual differences between specimens in courses, calibers, branching and merging patterns of cast vesical arteries, veins and capillaries.

### 3.2 Arterial supply

**FIGURE 2:**
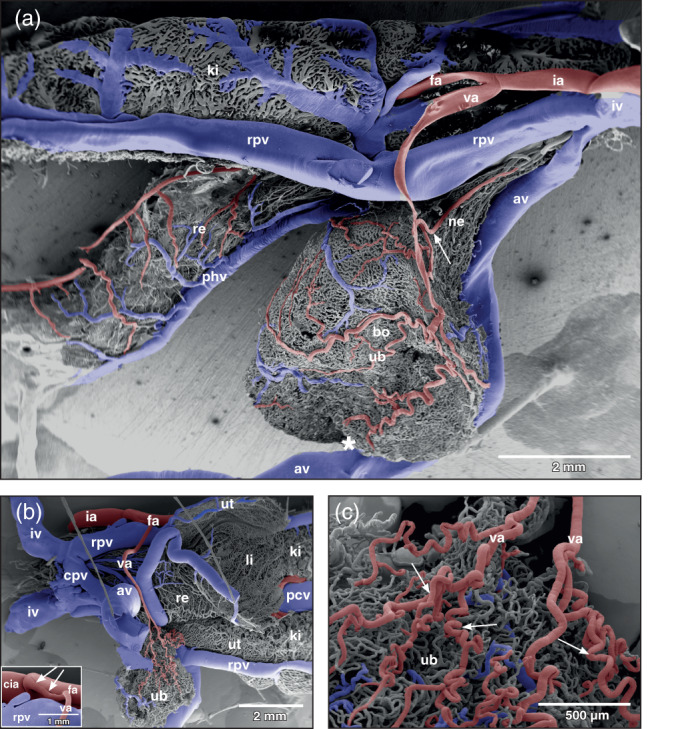
*Xenopus laevis*, vascular anatomy of the urinary bladder (ub) in an adult individual. (a) Gross arterial supply and venous drainage of a moderately distended (filled) bladder displaying a spherical body (bo) and a funnel‐like neck (ne). Vascular corrosion cast. Lateral view. Scanning electron micrograph. Anterior is to the left, dorsal is at top. Note the abdominal vein (av) which slightly notches the ventral bladder surface (asterisk). Arrow points at a lateral vesical artery supplying the bladder neck region. (b) Vascular anatomy of a contracted (emptied) bladder. Ventral view. The bladder is dislocated to the left side. Anterior is to the right. Origin, course, and branching pattern of the vesical artery (va) can be seen. Inset: Origin of the vesical artery (va) from the femoral artery (fa). Note the prominent flow divider (arrows). cia common iliac artery. (c) Coiling of vesical arteries (va) in a contracted bladder. av abdominal vein, cpv common pelvic vein, phv posterior hemorrhoidal vein, ia ischiadic artery, iv ischiadic vein, ki kidney, li large intestine, pcv posterior caval vein, re rectum, rpv renal portal vein, ut uterus (posterior portion of oviduct)

The arterial supply of the urinary bladder of adult *Xenopus laevis* was by a single vesical artery from either side. This artery branched off the proximal portion of the femoral artery (Figure 2(a),(b)) and showed a prominent flow divider at its origin (Figure 2(b), inset, arrows). The artery descended, crossed the renal portal vein laterally and coursed towards the lateral equatorial circumference of the bladder. Before it approached the serosal surface of the body of the bladder, it gave off a caudally directed branch (Figure 2(a), arrow). This branch ran along the ventro‐lateral surface of the neck of the bladder and gave off small branches that supplied ventral and ipsilateral neck regions (Figure 2(a)).

**FIGURE 3:**
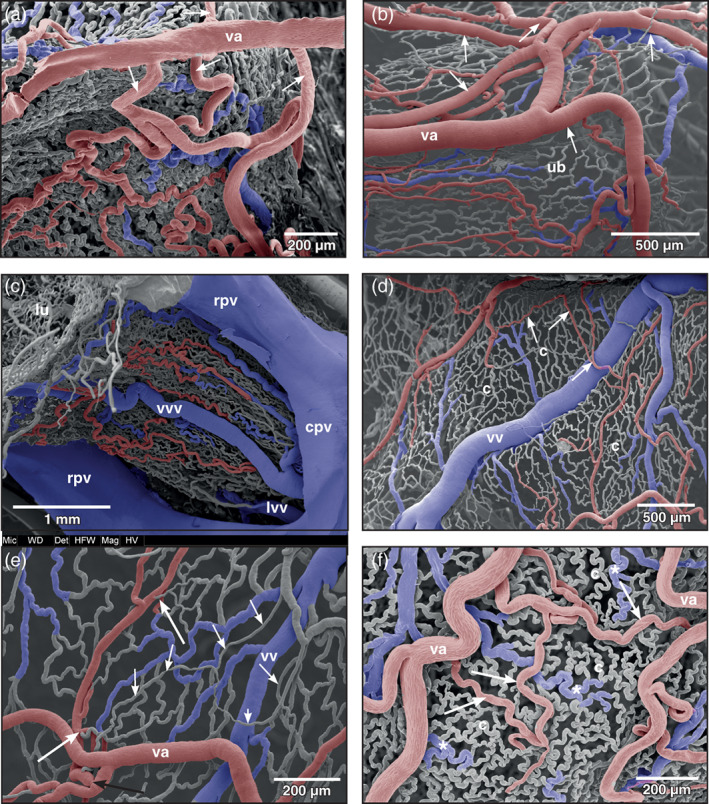
*Xenopus laevis*, branching patterns and courses of vesical arteries (va) as seen from the serosal side. (a) Branching pattern of the vesical artery in a moderately distended bladder. Anterior is at the top. Interbranching distances of the vesical artery (va) are small. Branches course towards the apex, the lateral surfaces, and the neck region of the bladder (arrows). (b) Same as (a) but in a stronger distended bladder. (c) Arterial and venous patterns of the ventral neck region of the bladder. (d, e) Microvascular bed of distended (filled) bladders. Terminal vesical arterioles feed single capillary‐sized vessels (short arrows) overlaying mucosal capillaries (c). Note different calibers and lengths of terminal arterioles (long white arrows) and an arterial sphincter (long black arrow) in (e). (f) Microvascular bed of a contracted (empty) bladder. Note slightly undulating vesical arteries (va) and terminal vesical arterioles (arrows). Strongly undulating capillaries (c) form small meshes. Postcapillary venules meander (asterisks). cpv common pelvic vein, lvv lateral vesical vein, lu lumen of urinary bladder, rpv renal portal vein, ub urinary bladder, vv vesical vein, vvv ventral vesical vein

**FIGURE 4:**
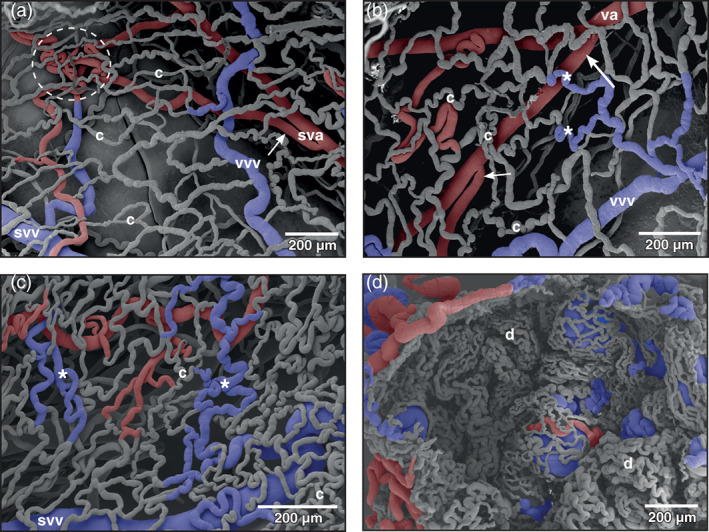
*Xenopus laevis*, patterns of mucosal vessels of the urinary bladder as seen from the mucosal side. (a) Distended bladder. Most mesh capillaries (c) are straight, a few capillaries only undulate. A maximum of three layers of vessels (capillaries, venules or small veins, arterioles, or small arteries) is seen. A local convolution of small vesical arteries (sva) is shown (encircled area). Arrow points at a bifurcation of a vesical artery with daughter vessels laying close aside. (b) Similar to (a), but less distended bladder displaying more undulating capillaries (c) and postcapillary venules (asterisks). Note small (short arrow) and large branching angles (long arrow) of a vesical artery (va). (c) Contracted (empty) bladder. Note strong undulating capillaries (c) and postcapillary venules (asterisks). (d) Strongly contracted bladder displaying narrow spaced mesh capillaries of dome‐shaped mucosal folds (d). svv small vesical vein, vvv vesical venule

At the lateral serosal surface the vesical artery splitted within a short distance into four to five smaller arteries (Figure 2(a)‐(c); Figure 3(a),(b); arrows). The larger arteries coursed anteriorly towards the apex of the bladder, the smaller ones ran posteriorly towards the neck region (Figure 3(a),(C)). In the emptied (contracted) bladder arteries which ran towards the apex of the bladder coiled strongly (Figure 2(c); arrows). In the moderately to heavily filled (distended) bladder these branches only undulated slightly or appeared as rather straight vessels. The arteries showed either Y‐shaped, blunt‐angled bifurcations with daughter vessels having almost the same diameters (Figure 4(b), long arrow) or gave off side‐branches with calibers smaller than that of the parent artery (Figure 4(a),(b); small arrow). At branching sites, sphincters were present (Figure 3(e), black arrow). Vesical arteries gave rise to thin short terminal arterioles (Figure 3(e), white arrows). Generally, terminal vesical arterioles ran over some distance without further branching. These arterioles then fed the capillary bed just beneath the transitional epithelium (Figure 3(f); arrows).

### 3.3 Capillary bed

In the moderately distended urinary bladder, capillary beds formed mesh works in which capillaries undulated only slightly (Figures 3(d),(e) and 4(a),(b)). Arteriolar‐capillary‐venular transition distances were locally quite different and varied from very short (Figure 3(e)) to moderately long (Figures 3(d),(f) and 4(a),(b)). In the contracted urinary bladder mesh capillaries undulated strongly and lay close aside each other (Figure 4(c)). In the strongly contracted bladder, mucosal capillaries lay side by side and formed dome‐like structures representing the mucosal foldings (Figure 4(d)).

Occasionally, single capillaries ran freely over a long distance before they drained into postcapillary venules (Figure 3(d),(e); short arrows).

### 3.4 Venous drainage

**FIGURE 5:**
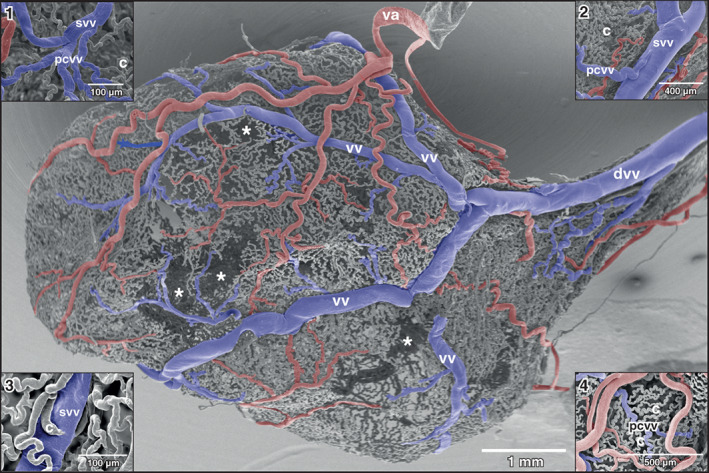
*Xenopus laevis*, drainage of dorsal and dorso‐lateral areas of the urinary bladder. Dorsal view at the bladder shown in Figure 2(a) *. anterior* is to the left. Asterisks mark wall areas where incomplete filling of the vasculature occurred. Note four vesical veins (vv) which form the dorsal vesical vein (dvv). Va vesical artery. Inset 1: Formation of a small vesical venule (svv) from postcapillary venules (pcvv). Inset 2: Postcapillary venule (pcvv) draining into a small vesical venule (svv). Note the relation of the calibers of the venules. Inset 3: Capillary (c) draining into a small vesical vein (svv). Inset 4: Undulating capillaries (c) drain in series bilaterally into a postcapillary venule (pcvv)

**FIGURE 6:**
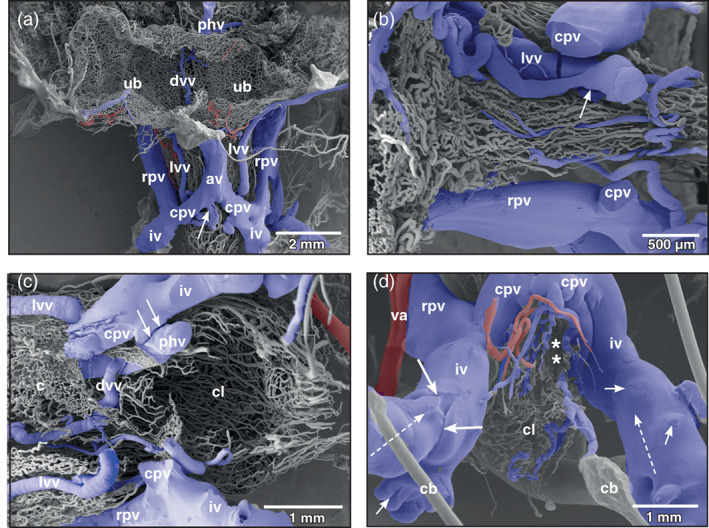
*Xenopus laevis*, venous drainage of the urinary bladder (ub). (a) Ventro‐caudal view at the large veins running aside the neck region of the bladder. Incomplete specimen. Anterior is at the top, posterior is at the bottom. A lateral vesical vein (lvv) is seen to join the right common pelvic vein (cpv) at its caudal aspect (arrow). (b) Vascular anatomy of the ventral neck region of the bladder after removal of the common pelvic veins (cpv). Anterior is at the left, posterior is to the right. A lateral vesical vein (lvv) is joined by a smaller one (arrow) to finally drain into right or left common pelvic vein (not shown). (c) Vascular anatomy of the cloacal region. Only remnants of the mucosal capillary bed (c) of the dorsal wall of the bladder are left. Note the dorsal vesical vein (dvv) which drains via the posterior hemorrhoidal vein (hv) into the left common pelvic vein (cpv). At its entrance at the caudal margin of the common pelvic vein, the posterior hemorrhoidal vein is broken (arrows). (d) Venous valves of the ischiadic veins (iv). Ventro‐caudal view. In the orthogradely filled large valve, the leaflet structure is clearly replicated (long arrows). Note small valves where retrograde resin flow was stopped at the valves (short arrows). Dashed arrows mark direction of blood flow, asterisks mark slit‐like opening of bladder into cloaca. av abdominal vein, cb conductive bridge, cl cloaca, rpv renal portal vein, va vesical artery

The mergings of several small postcapillary venules to a larger venule occurred within a short distance (Figure 5, inset 1). In some areas, large postcapillary venules joined larger vesical veins in an obtuse angle (Figure 5, inset 2). Occasionally, also capillary‐sized vessels drained into larger veins (Figure 5, inset 3). Small vesical venules displayed two basic patterns. In most cases they formed by merging of postcapillary venules of different sizes (Figures 3(d),(e), 4(a)–(c), and 5). In some specimens larger postcapillary venules received regularly spaced capillaries bilaterally (Figure 5, inset 4). Vesical veins ascended from lateral and ventral serosal surfaces towards the dorsal surface. Course, calibers and merging patterns of vesical veins varied between individual specimens (Figure 5). Generally, they joined at the transitional area of the bladder body into the neck region and formed a single large dorsal vesical vein (Figure 5). This vein coursed caudally along the dorsal serosal surface of the neck region and emptied into the posterior hemorrhoidal vein (Figure 6(a)‐(c), large arrows). Additionally, smaller veins coursed along the serosal surfaces of the bladder neck region in a caudal direction (Figures 3(c) and 6(a)) and drained into right or left common pelvic veins (Figure 6(a), arrow). Common pelvic veins interconnected with ischiadic veins (Figures 2(b), 3(c), and 6(a)‐(d)) and drained into the abdominal vein (Figures 2(a) and 6(d)). Ischiadic veins possessed prominent venous valves, which were filled orthogradely (Figure 6(d), long arrows) or retrogradely (Figure 6(d), short arrows). Ischiadic veins continued rostrally as renal portal veins (Figures 2(b), 3(c), and 6(a)‐(d)).

## 4 DISCUSSION

In the present study, no specific pretreatment of animals was performed to gain a defined filling state of the urinary bladder. The filling state was estimated from casted specimens. Large casted bladders with only slightly undulating vesical arteries and wide capillary meshes were considered filled (distended), smaller ones with strongly undulating vesical arteries and narrow capillary meshes were considered empty (contracted). Out of eight vascular castings three specimens presented distended bladders (1 female, 2 males), one specimen possessed a moderately distended bladder (female), and four specimens had an empty (contracted) bladder (1 female, 3 males). No obvious differences in the vascular anatomy of the bladders of female and male *Xenopus* were found. The grade of filling of the urinary bladder vasculature in the castings differed with no obvious dependence from filling states of urinary bladders and sexes. If one considers the thin wall of the urinary bladder in combination with a proposed intraluminal pressure in a highly filled (distended) bladder—for the aquatic frog *Lithobates catesbeiana* a mean internal bladder pressure of 0,16 kPa was reported (Martin & Hillman, 2009)—it is not surprising that only one out of eight vascular castings via the arterial trunk showed an almost complete filled vascular bed with only few missing microvascular areas.

Our study confirmed the findings of Millard (1940) that in *Xenopus laevis* one vesical artery branched off the femoral artery bilaterally close to its origin from the common iliac artery. This contrasts with the situation in *Rana esculenta* (Gaupp, 1904), and *Pelobates fuscus*, *Bombina bombina* and *Bufo bufo* (Szarski, 1948) where the vesical artery branches off the epigastric artery, a branch of the femoral artery. *Xenopus* lacks an epigastric artery.

Of interest was the pronounced flow divider found in several specimens at the origin of the vesical artery from the femoral artery. This flow divider by its orientation efficiently governed blood flow into the rather long vesical artery and thus it was considered an important structure to ensure the blood supply of the urinary bladder under varying filling conditions of the bladder. Of interest was also that vesical arteries ran unbranched for a long distance behaving as conducting arteries before they changed into supplying arteries, which give off branches within much shorter distances (see Figure 3(a),(b)). The arrival and the branching patterns of the vesical arteries at the lateral serosal surface of the bladder and their coilings are considered to be of functional importance, as they will allow a sufficiently high blood flow when the urinary bladder fills or empties. A ventral position of the arriving vesical arteries most likely would have compromised bladder blood supply when the bladder was totally filled (distended) with urine, which by its weight would compress arteries located there. Which effect a filled (distended) urinary bladder exerted upon the abdominal vein, which ran along the ventral circumference of the body of the bladder (Figures 1(a),2(a)) is not studied yet. Assuming that in the moderately filled bladder vesical veins in general drained via common pelvic veins into the abdominal vein, any pressure exerted by a highly filled bladder upon the abdominal vein might have changed the drainage from the common pelvic veins towards the renal portal veins. No venous valves were found in the pelvic veins that could have prevented this route. Blood from the urinary bladder therefore could, at least in part, drain towards the kidneys instead of the liver and so establish a humoral communication between urinary bladder and kidney. If this situation actually occurs under the conditions proposed, it yet has to be proven.

Anatomical structures reported to control blood flow in the urinary bladder are venous valves and arterial sphincters. Abundant venous valves within the bladder microvasculature have been reported in rat, guinea pig, rabbit, dog, and pig (Hossler & Kao, 2007; Hossler & Monson, 1995, 1998a, 1998b). Arterial sphincters have been described in dog and pig only (Hossler & Kao, 2007). In the urinary bladder of *Xenopus*, arterial sphincters were also present and most likely participated in the regulation of blood flow towards the areas of need.

In urinary bladders of many mammals, collateral circulations have been described, particularly between the main supply vessels at the serosal surface and within the mucosal vascular bed (Hossler & Kao, 2007). In the urinary bladder of *Xenopus* few arterio‐arterial anastomoses were found. Arterial branching modes and vessel courses were obviously sufficient to ensure blood supply.

In our opinion, the two patterns of terminal vesical arterioles, i.e. short and long, most likely serve the flexibility of blood flow in the mucosa under varying filling conditions. While the long arterioles allow greater spatial movements of the mucosa, the short arterioles were thought (i) to establish short arteriolar‐capillary transition times in all states of bladder filling and (ii) to anchor the mucosal capillary bed to the vesical arterial system.

Of interest to mention are the few single, rather straight running capillary‐sized vessels found as the most abluminal located vessels (Figure 3(D),(e)). They most likely supplied bundles of vascular smooth muscle cells, which were too large to be supplied by diffusion from the mucosal capillary bed.

The formation of postcapillary venules varied greatly. Generally, capillaries formed small postcapillary venules, which gradually merged to form larger venules, small veins and vesical veins that emptied into the common pelvic veins. In some cases, however, capillaries emptied into veins having a much larger caliber, in others capillaries emptied bilaterally in series into a small postcapillary venule. The observation that postcapillary venules drained in an obtuse angle into a vein of much larger diameter is of interest. This mode of merging possibly builds up some backpressure, which most likely prolongs the arteriolar‐capillary‐venular transition time and so provides for a better blood supply to the mucosa.

In *Xenopus laevis*, ureters do not open into the urinary bladder but they open at the urogenital papilla (in males) or at the urinary papilla (in females) into the cloaca (Brown, 1970). The mode of filling of the urinary bladder therefore is quite different from that of mammals in which ureters open directly into the bladder and in which a valve at the bladder entrance prevents reflux of urine back into the ureter (Hossler & Kao, 2007). According to Martin and Hillman (2009) who studied the terrestrial toad, *Chaunus marinus* and the aquatic frog, *Lithobates grylio* “..bladder filling is a result of pressures generated in the ureters and the cloaca that are greater than internal bladder pressures and that the more aquatic species had a less compliant urinary bladder compared with that of the terrestrial species”. Our observations that the urinary bladder of *Xenopus laevis* was highly distended in some specimens indicated that its bladder is compliant. Obviously, the maximally four layers of blood vessels within the thin wall of the organ by their courses and patterns easily follow the dimensional changes of the bladder during distention by changing from highly coiled patterns of arteries, capillaries and veins in contracted (emptied, slightly filled) bladders to slightly undulating (in moderately filled) or even straight courses in distended (filled) bladders.

## AUTHOR CONTRIBUTIONS

**Alois Lametschwandtner:** Conceptualization; investigation; methodology; visualization; writing‐original draft; writing‐review and editing. **Bernd Minnich:** Conceptualization; methodology; resources; visualization; writing‐original draft; writing‐review and editing.

## CONFLICT OF INTEREST

The authors have no conflict of interests to declare.

### PEER REVIEW

The peer review history for this article is available at https://publons.com/publon/10.1002/jmor.21310.

## Data Availability

All corrosion cast and histological samples are stored a the Department of Bioscineces, University of Salzburg. The material is available at request from the corresponding author.
